# Macro- and microscopic anatomy of the digestive tract in the red-eared slider (Emydidae: *Trachemys scripta elegans*)

**DOI:** 10.1371/journal.pone.0315737

**Published:** 2024-12-30

**Authors:** Nonoha Miyai, Takuma Kozono, Tatsu Kuriki, Mai Todoroki, Tomoaki Murakami, Kyosuke Shinohara, Toshinori Yoshida, Tetsuhito Kigata

**Affiliations:** 1 Cooperative Department of Veterinary Medicine, Tokyo University of Agriculture and Technology, Tokyo, Japan; 2 Smart-Core-Facility Promotion Organization, Tokyo University of Agriculture and Technology, Tokyo, Japan; 3 Laboratory of Veterinary Pathology, Tokyo University of Agriculture and Technology, Tokyo, Japan; 4 Laboratory of Veterinary Toxicology, Tokyo University of Agriculture and Technology, Tokyo, Japan; 5 Department of Biotechnology and Life Science, Tokyo University of Agriculture and Technology, Tokyo, Japan; 6 Laboratory of Veterinary Anatomy, Tokyo University of Agriculture and Technology, Tokyo, Japan; Cairo University, Faculty of Science, EGYPT

## Abstract

The red-eared sliders (Emydidae: *Trachemys scripta*) is characterised by a high adaptability to a variety of environment and threatens the habitat of Japanese native species. The ability to digest a variety of diets may attribute to the high adaptive capacity of this species to various environments, however, the digestive morphology remains scarcely described in red-eared sliders. In this study, we investigated the macro- and microscopic anatomy of the esophagus, stomach, small intestine, and large intestine in red-eared sliders. All segments of the digestive tract had longitudinal mucosal folds, the height and width of which varied in each segment of the digestive tract. The stomach had the highest and widest mucosal folds. The mucosal folds in the proximal-to-middle small intestine exhibited a zigzag shape, whereas those in the distal small intestine were linear. The wall of the digestive tract regularly consisted of mucosa, submucosa, tunica muscularis, and tunica adventitia or serosa. In each segment of the digestive tract, the epithelial structure was different. The esophagus and small intestine were lined by the pseudostratified columnar epithelium. In both segments, the basal part of the pseudostratified epithelium included proliferating cell nuclear antigen (PCNA)-positive proliferating cells. The stomach and large intestine were lined by the simple columnar epithelium. In the stomach and large intestine, PCNA-positive proliferating cells were present in the neck of the proper gastric gland and crypt-like structures, respectively. The proper gastric gland was composed of oxynticopeptic and mucous cells. This study revealed the detailed macro- and microscopic anatomy of the digestive tract in red-eared sliders. Overall, our findings may provide an anatomical basis for understanding the relationship between morphology and function in the digestive tract of turtles.

## Introduction

Turtles are widely distributed in freshwater, marine, and terrestrial environments and occupy various trophic niches [1, 2]. Adapting to their respective habitats, turtles show a variety of feeding habits, such as the carnivore, herbivore, and omnivore [3, 4]. The digestive tract is structured for efficient digestion and absorption of nutrients from various diets of each species [5–7]. In terms of anatomy, for example, the digestive tract generally has mucosal folds and villus in the internal surface to increase the absorptive efficiency [8, 9]. These structures are lined by the epithelial cells with various compositions, which differ according to their segment-specific functions, such as absorption and secretion [10]. Moreover, to maintain the normal epithelial structure and its function, the epithelial progenitor cells locate in the crypts and renew the epithelial cells constantly [11, 12].

The mucosal fold and epithelial structure exhibits distinctive features in each turtle species [13–15]. For example, in the esophagus, tortoises (family Testudinidae) have longitudinal folds lined by non-keratinised stratified squamous epithelium and esophageal glands, whereas sea turtles (family Cheloniidae) have papillae lined by keratinised stratified squamous epithelium and no esophageal glands [16, 17]. In Podocnemididae, the proximal and distal portions of the esophagus have papillae and longitudinal folds, respectively. These structures are lined by the non-keratinized stratified squamous and stratified columnar [13]. The small intestine exhibits long and narrow longitudinal folds in Greek tortoises [16], a zigzag or reticular folds in green sea turtles and Podocnemididae turtles [13, 14, 18] and a honey-combed shape in leatherback turtles [18]. Testudinidae and Cheloniidae differ in their biological characteristics, such as habitat and diet, and these factors have been suggested to influence the digestive tract morphology of the turtles [13, 18]. To understand the relationship between the morphology of digestive tract and biological features, further investigations are required in a greater number of species having various biological features. It is also conceivable that the proliferating cells distribution, which may show a turtle-specific pattern corresponding to the characteristic mucosal folds and epithelial cell structures, would be an important insight into understanding the mechanisms to maintain the normal epithelial morphology in turtles. However, previous studies have described the morphology of the mucosal folds and epithelium without any description of the proliferating cell distribution.

The family Emydidae includes species with a variety of feeding habits [19]. However, a comprehensive investigation of the digestive tract morphology has not yet been conducted in any species of this family. The red-eared slider (Emydidae: *Trachemys scripta elegans*) is an omnivorous freshwater turtle and is characterized by an adaptability to a variety of ecological environment [19, 20]. Previously reported species had low dietary diversity [21, 22]. For example, the Egyptian tortoise eats only plant [21], the green sea turtle mainly eats sea glasses and sea weeds [22]. In contrast, the red-eared slider can eat various foods, such as terrestrial and water snails, insects, crustacea, fish and plants [20, 23, 24]. The ability to feed on a wide variety of animals and plants a characteristic of this species and is thought to be one of the factors enabling its very widespread distribution. In Japan, the red-eared slider threaten native ecosystems by depriving native species of food and eating water plants and aquatic organisms [24]. This ecology may be underlain by the unique digestive morphology of red-eared slider. Therefore, in the present study, we aimed to investigate the macro- and microscopic anatomy of the digestive tract in the red-eared slider to provide an anatomical base for better understanding of the relationship between the morphology and function of the digestive tract in turtles.

## Materials and methods

### Animals

**Fig 1:**
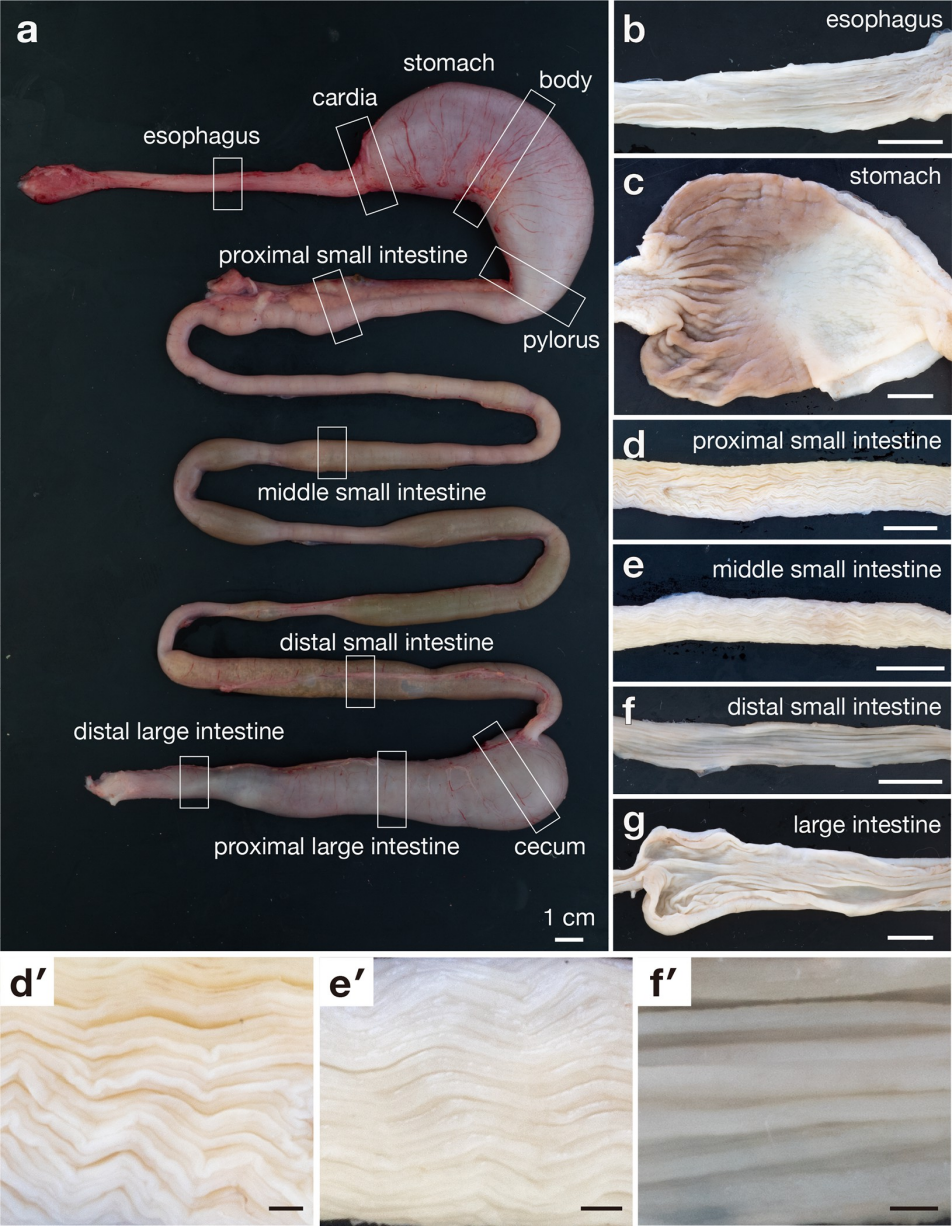
Photographs of the unfolded digestive tube (a) and the mucosal folds of each segment of the digestive tract (b-g). (a) shows the unfolded digestive tract with blocks that indicate the sampling sites. (b)-(g) show the mucosal fold of the esophagus (b), stomach (c), proximal small intestine (d), middle small intestine (e), distal small intestine (f), and large intestine (g). (d′)-(f′) show the enlarged view of the mucosal folds if proximal, middle, and distal small intestine, respectively. Scale bar = 1 cm.

All animal experimental procedures conducted in the present study were approved by the Research Ethics Committee for Animal Experimentation of the Tokyo University of Agriculture and Technology (Tokyo, Japan). Seven female red-eared sliders (*T*. *scripta elegans*) collected in Tokyo, weighing 1.0–1.4 kg, were used in this study. The animals were euthanised with sodium pentobarbital (over 100 mg/kg, intracoelomically or intracardially) [25]. The plastron was removed using an electric cast cutter (CB-06; YDM Corporation, Tokyo, Japan) to harvest the digestive tube from the esophagus to the colon (Fig 1).

### Gross anatomy

The internal appearance of two turtles was investigated. The digestive tract was opened longitudinally on the antimesenteric side, washed gently with phosphate-buffered saline (PB), and content residues thereafter removed. The digestive tract was stretched on a rubber board and photographed using a digital camera (Nikon D5500; Nikon Imaging Japan Inc., Tokyo, Japan) (Fig 1). The colour, sharpness, brightness, and resolution of the images were adequately adjusted using Adobe Photoshop (Adobe Systems, San Jose, CA, USA). Subsequently, the images were assembled in Adobe Illustrator CC (Adobe Systems). The light and electron micrographs (described below) were also processed with these applications.

### Light microscopic observation

In four turtles, samples with a 1 cm length along the oro-anal axis were collected at the following sites for each segment: esophagus, stomach, proximal small intestine, middle small intestine, distal small intestine, caecum, proximal colon, and distal colon (Fig 1). Immediately after the samples were gently washed with PB to remove content residues, they were fixed with 10% formaldehyde in 0.1 M PB. Subsequently, the samples were embedded in paraffin and cut transversely or longitudinally at a 3 μm thickness. Sections were stained with conventional haematoxylin and eosin to examine the general laminar structure. To observe mucin-producing cells, additional sections were stained with alcian blue (AB) pH 2.5, or periodic acid Schiff (PAS), or combined AB and PAS (AB-PAS) followed by nuclear fast red (in AB staining) or haematoxylin (in PAS and AB-PAS staining). Digital images of representative sections were obtained using a light microscope (BZ-9000; Keyence Corporation, Osaka, Japan).

### Immunohistochemistry

The paraffin-embedded tissues were subjected to immunohistochemistry. Antigen retrieval was performed using 0.01 M citrate buffer (pH 6.0) at 90°C for 10 min using a microwave. Then, all sections were incubated in a blocking solution (PBS containing 1.5% normal horse serum) for 30 minutes. Subsequently, sections were applied to primary antibodies against proliferating cell nuclear antigen (PCNA) (1:200 dilution, Agilent Technologies, Ltd., Santa Clara, CA, USA) using Vectastain Elite ABC-HRP kits (Vector Laboratories, Inc., Newark, CA, USA). Positive signals were visualised with DAB (3,3′-diaminobenzidine), and the sections were stained with haematoxylin.

### Electron microscopic observation

The collected tissues were cut into segments (~1 cm), fixed with 4% glutaraldehyde (GA) (#079–00533; FUJIFILM Wako Pure Chemical, Osaka, Japan) in PB at pH 7.2 (#160–14481; FUJIFILM Wako Pure Chemical) overnight, and washed with PB.

To prepare the scanning electron microscopy (SEM) samples, the tissues were post-fixed with 1% OsO_4_ (#300; Nisshin EM, Tokyo, Japan) for 4 h. The fixed tissues were then dehydrated in a graded ethanol series (#057–00451; FUJIFILM Wako Pure Chemical) and substituted with *t*-butyl alcohol (#025–03396; FUJIFILM Wako Pure Chemical). After freeze-drying, the tissues were mounted onto brass specimen stubs using carbon adhesive tabs and silver paste (#712; Nisshin EM), osmium-coated using a coater (Neoc-STB; MEIWAFOSIS Co., Ltd., Tokyo, Japan), and observed via SEM (JSM-7100F; JEOL Ltd., Tokyo, Japan) at 1.50 kV.

To prepare the transmission electron microscopy (TEM) samples, GA-fixed tissues were cut into small pieces (1~8 mm^3^) and post-fixed with 1% OsO_4_ for 1 h. After dehydration in a graded ethanol series, the tissues were substituted with propylene oxide (#311; Nisshin EM) and embedded in Epon812 (T024; TAAB, Berkshire, UK). Ultrathin sections of 70-nm thickness were prepared using an ultramicrotome (Leica EM UC7; Leica Microsystems, Tokyo, Japan) and placed on Formvar-coated copper grids. The sections were contrasted with an EM stainer (#311; Nisshin EM) and lead citrate, and thereafter observed using TEM (JEM-1400Flash; JEOL Ltd.) at 100 kV. Stomach sections were placed on a SiN Window Chip (#783131836; JEOL Ltd.) and contrasted, as described above. To acquire ultra-widefield montage images, the Limitless Panorama system, a software for automatic montage systems on TEM, was used.

### Morphometry of mucosal folds

The slides were scanned with a virtual slide system BX61VS (Olympus corporation, Tokyo, Japan) linked with VS-ASW (Ver. 2.8; Olympus corporation), and the hight and width of the mucosal folds were measured in all mucosal folds in each segment of four turtles. The number of mucosal folds measured were as follows: 36 from esophagus, 24 from stomach, 190 from proximal small intestine, 154 from middle small intestine, 34 from distal small intestine, 31 from cecum, 25 from proximal large intestine, and 38 from distal large intestine.

### Statistical analysis

The data were analyzed using the Kruskal–Wallis test for each segment, followed by Steel–Dwass multiple comparison tests using the software JMP 18 (SAS Institute, Cary, NC, USA). A *p* value of <0.01 was considered statistically significant.

## Results

### Internal macroscopic morphology of the digestive tract

Based on its external appearance, the digestive tract was divided into the esophagus, stomach, small intestine, caecum, and large intestine (Fig 1A). All segments of the digestive tract had longitudinal mucosal folds (Fig 1B to 1G). The mucosal fold in the proximal and middle small intestines exhibited a zigzag shape (Fig 1D, 1D′, 1E, 1E′), whereas those in other segments were linear (Fig 1B, 1C, 1F, 1F′, 1G).

**Fig 2:**
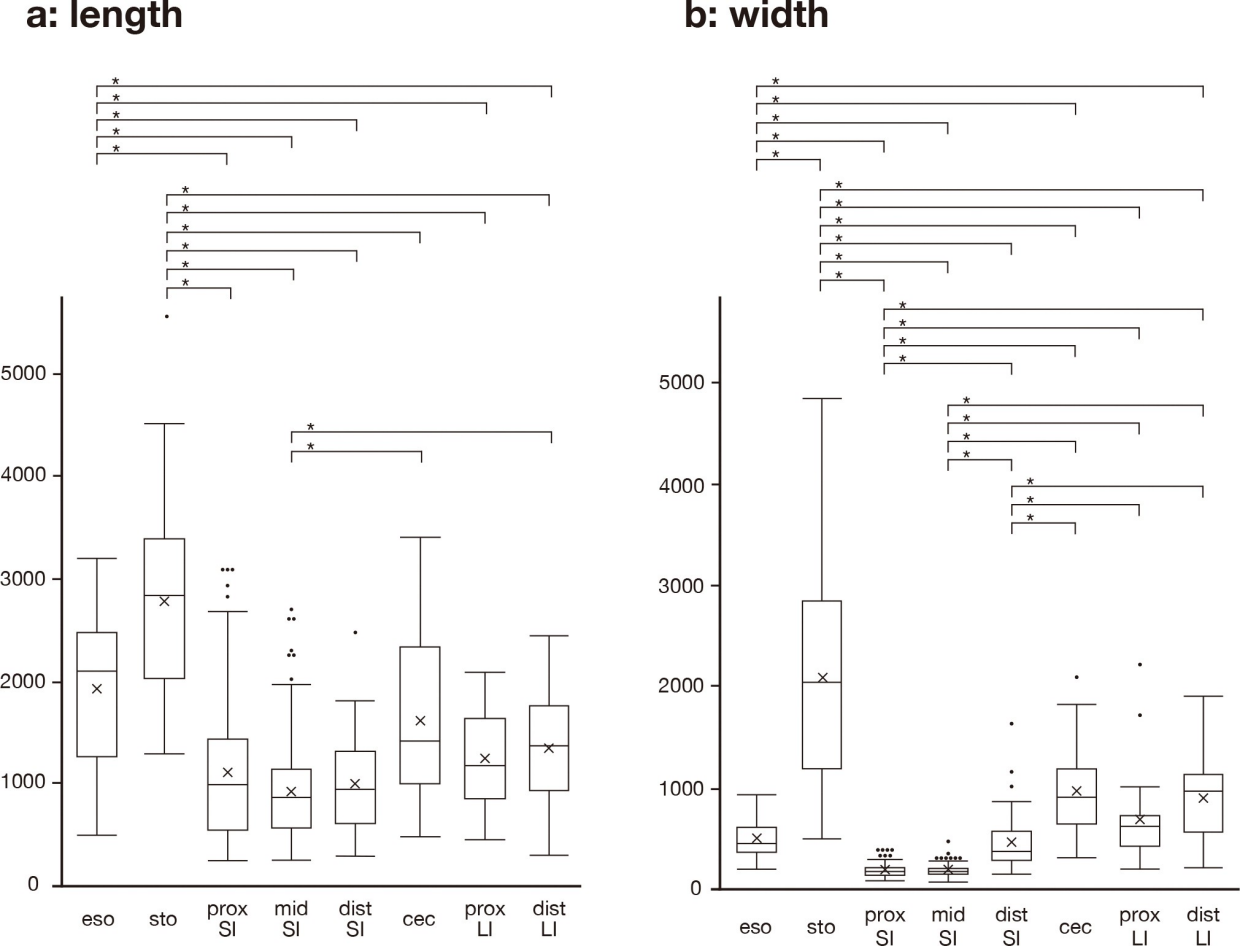
Box plots showing the statistical difference in the length (a) and width (b) of the mucosal fold in each segment of digestive tract. In each box, horizontal line indicates median value, box extend from 25% to 75% of distribution of values in each digestive segment, vertical extending lines indicate adjacent values, dots indicate outlier, and cross marks indicate mean value. *, *P*<0.01. eso, esophagus; sto, stomach; prox SI, proximal small intestine; mis SI, middle small intestine; dist SI, distal small intestine; cec, cecum; prox LI, proximal large intes tine; dist LI, distal large intestine.

The hight and width of the mucosal folds increased from the esophagus to the stomach (Figs 1B, 1C and 2). The mucosal fold in the stomach was the highest and widest among all sections in the digestive tract (Figs 1C and 2). The height of the mucosal folds decreased in the segments distal to the stomach (Figs 1C–1G and 2). The width of mucosal folds was gradually increased from the middle small intestine to large intestine (Figs 1E–1G and 2).

### Microscopic morphology of the digestive tract wall

**Fig 3:**
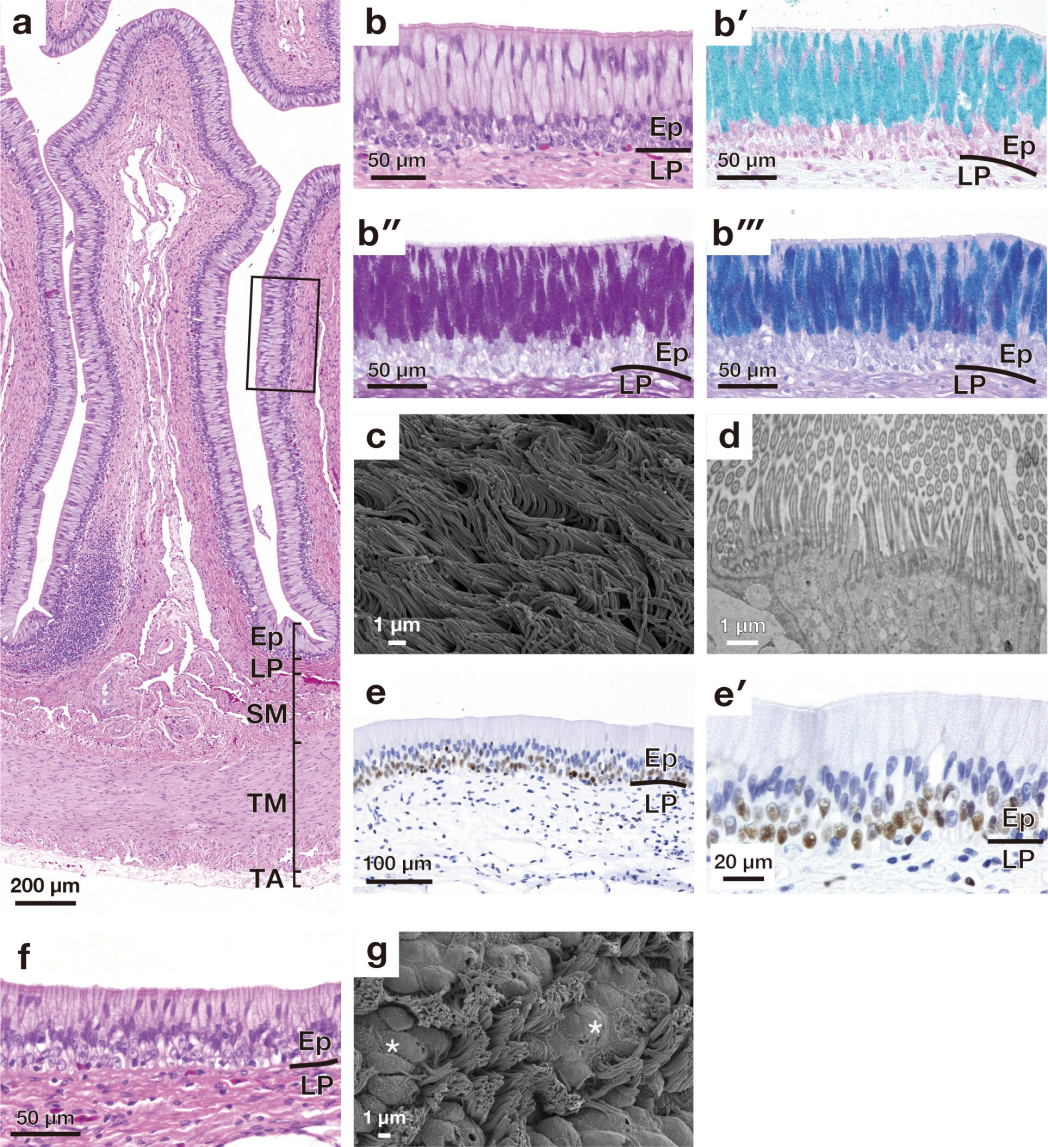
Microscopic appearance of a mucosal fold in the esophagus and esophago-gastric junction. (a) Overall cross-sectional view of a longitudinal fold stained with haematoxylin and eosin (HE). (b) shows a higher magnification of the mucosal epithelium enclosed by solid lines in (a). (b′)-(b‴) show high magnification images of the epithelial layer stained with alcian blue (AB) (b′), periodic acid Schiff (PAS) (b″), combined AB and PAS (b‴). (c) is scanning electron microscopy (SEM) image of the mucosal epithelium in the esophagus. (d) is transmission electron micrograph showing the cilia. (e) shows proliferating cell nuclear antigen-positive cells present in the basal part of the epithelium. (e′) shows a high magnification image of PCNA-positive cells. (f) shows cross-section of the epithelium of the esophago-gastric junction stained with HE. (g) SEM image of the mucosal epithelium in the esophago-gastric junction. Asterisks indicate the goblet cells. Ep, epithelium; LP, lamina propria; SM, submucosa; TM, tunica muscularis; TA, tunica adventitia.

**Fig 4:**
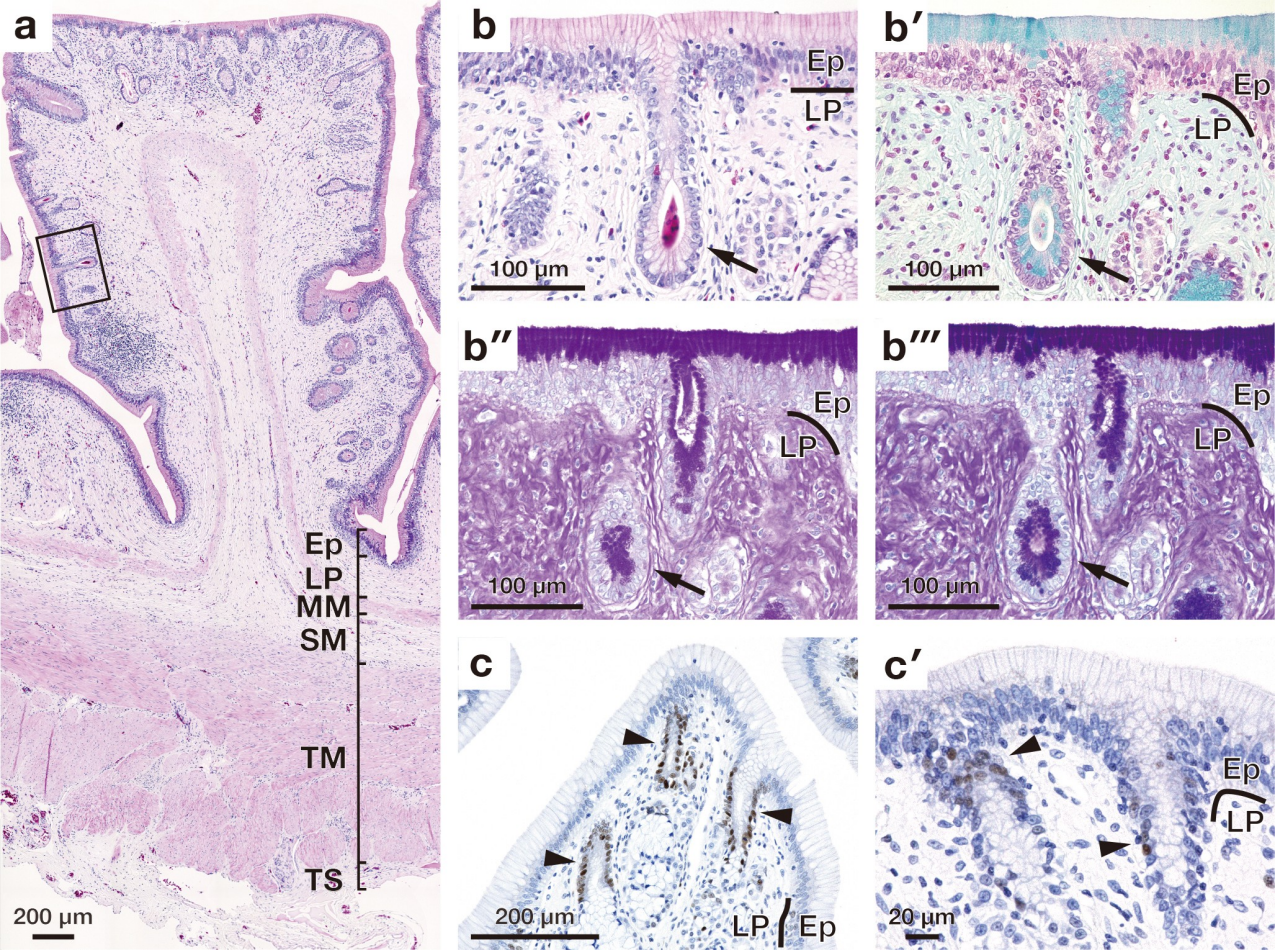
Microscopic appearance of the gastric fold in the cardia. (a) Overall cross-sectional view of the gastric fold stained with haematoxylin and eosin. (b) Higher magnification image of the mucosal epithelium enclosed by solid lines in (a). Arrow indicates the cardiac gland. (b′)-(b‴) show high magnification images of the epithelial layer stained with alcian blue (AB) (b′), periodic acid Schiff (PAS) (b″), combined AB and PAS (b‴). Arrow indicates the cardiac gland. (c) shows proliferating cell nuclear antigen (PCNA)-positive cells present in the basal part of the epithelium (indicated by arrowheads). (c′) shows a high magnification image of PCNA-positive cells. Arrowheads indicate PCNA positive cells. Ep, epithelium; LP, lamina propria; MM, muscularis mucosae; SM, submucosa; TM, tunica muscularis; TS, tunica serosa.

**Fig 5:**
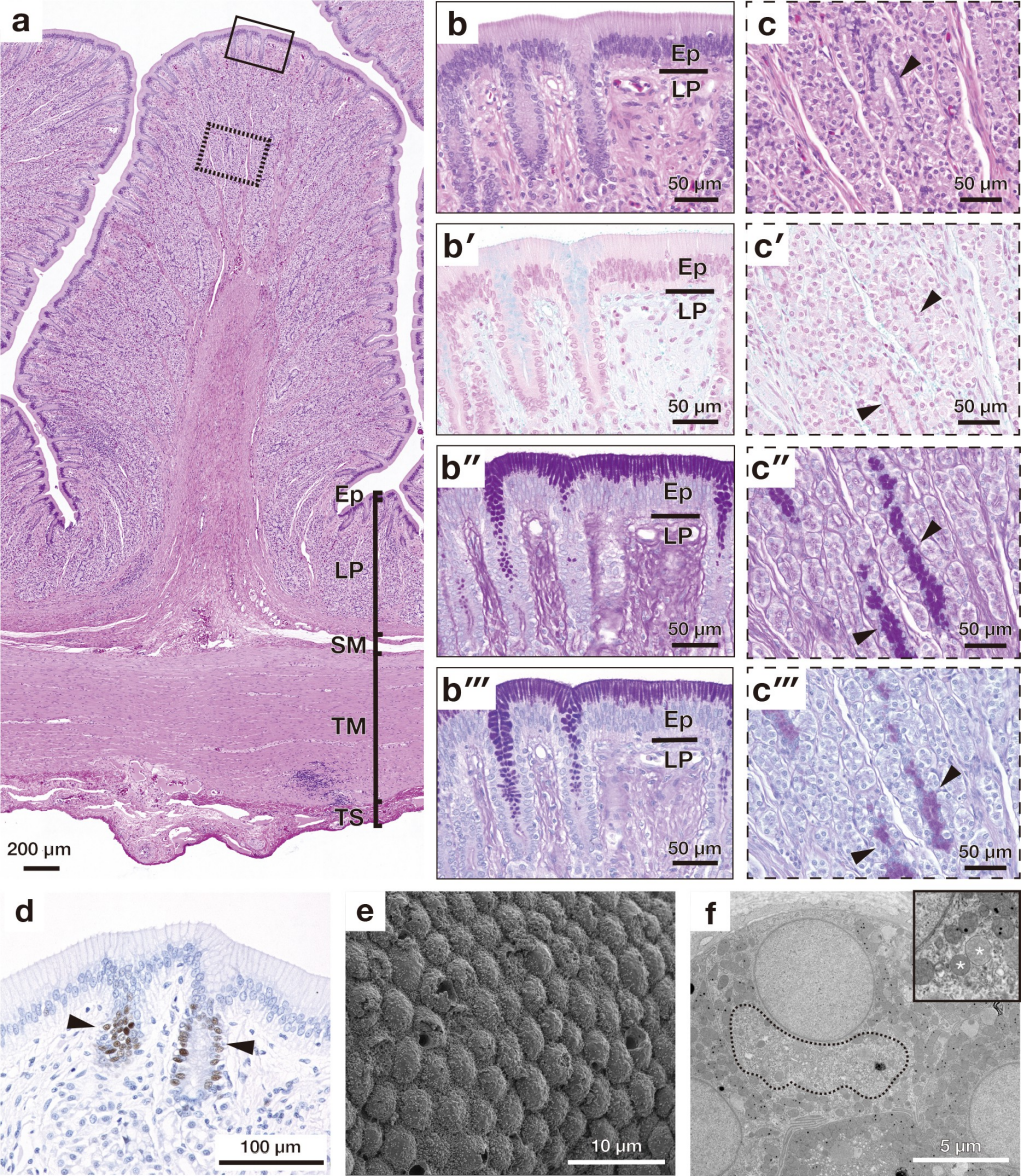
Microscopic appearance of the gastric fold in the body of stomach. (a) Overall cross-sectional view of the gastric fold stained with haematoxylin and eosin. (b) Higher magnification image of the mucosal epithelium enclosed by solid lines in (a). (b′)-(b‴) show high magnification images of the epithelial layer stained with alcian blue (AB) (b′), periodic acid Schiff (PAS) (b″), combined AB and PAS (b‴). (c) Higher magnification image of the gastric gland enclosed by broken lines in (a). (c′)-(c‴) show high magnification images of the gastric gland stained with alcian blue (AB) (c′), periodic acid Schiff (PAS) (c″), combined AB and PAS (c‴). Arrowheads indicate the mucus cells in the gastric gland. (d) shows proliferating cell nuclear antigen-positive cells present in the neck of the gastric gland. (e) Scanning electron micrograph of the mucosal epithelium. (f) Transmission electron micrograph of an oxynticopeptic cell. The secretory canaliculus locates in the area enclosed by the broken line. Inset shows a high magnification view of the secretory granule (indicated by asterisk) and mitochondria. Ep, epithelium; LP, lamina propria; SM, submucosa; TM, tunica muscularis; TS, tunica serosa.

**Fig 6:**
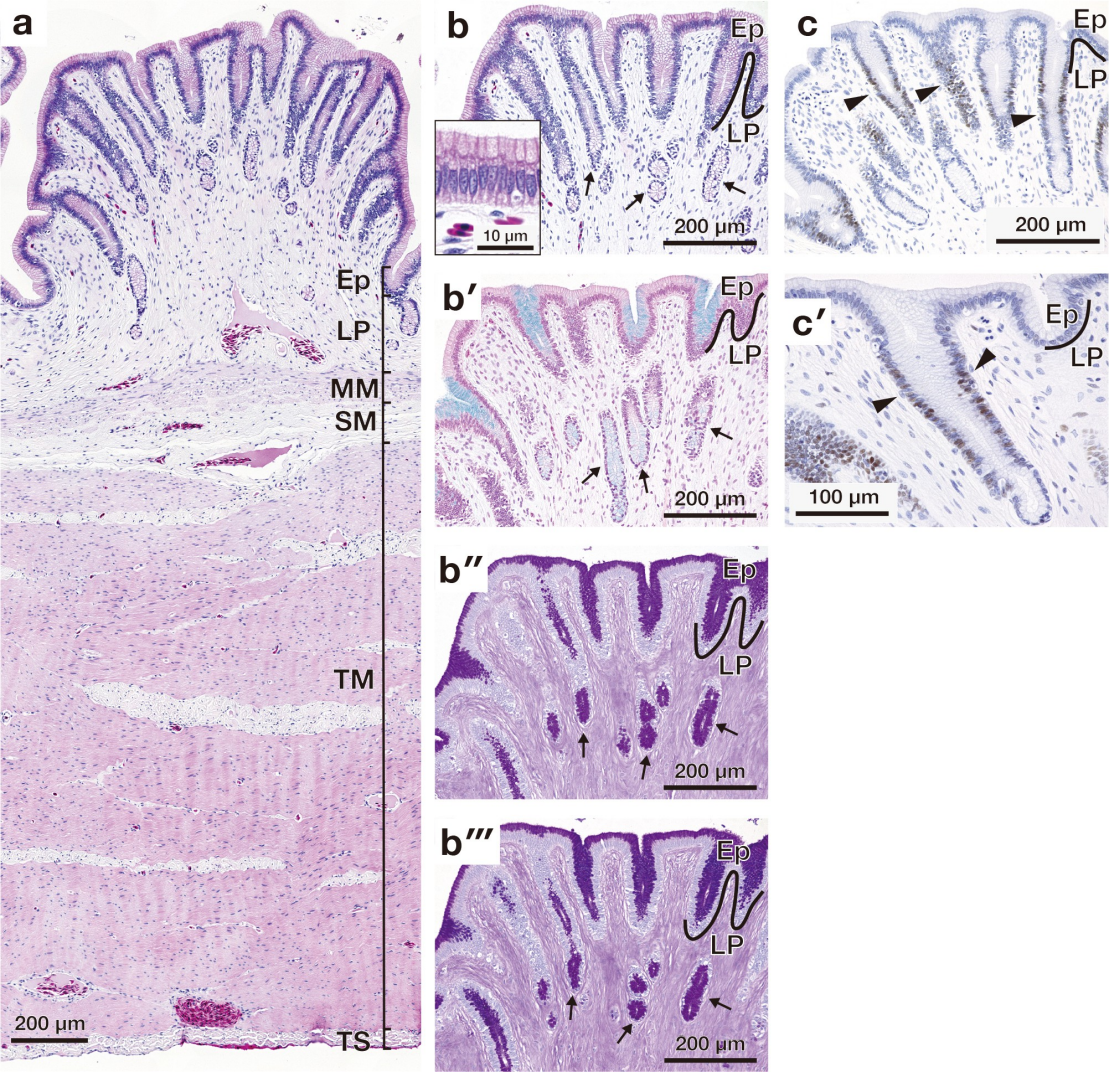
Microscopic appearance of the gastric fold in the pylorus. (a) Overall cross-sectional view of the gastric fold stained with haematoxylin and eosin. (b) Higher magnification image of the mucosal fold. Arrows indicate the pyloric gland. Inset shows higher magnification image of the epithelium of the mucosal fold. (b′)-(b‴) show high magnification images of the mucosal fold stained with alcian blue (AB) (b′), periodic acid Schiff (PAS) (b″), combined AB and PAS (b‴). Arrows indicate the pyloric gland. (c) shows proliferating cell nuclear antigen (PCNA)-positive cells present in the basal part of the epithelium (indicated by arrowheads). (c′) shows a high magnification image of PCNA-positive cells. Arrowheads indicate PCNA positive cells. Ep, epithelium; LP, lamina propria; MM, muscularis mucosae; SM, submucosa; TM, tunica muscularis; TS, tunica serosa.

**Fig 7:**
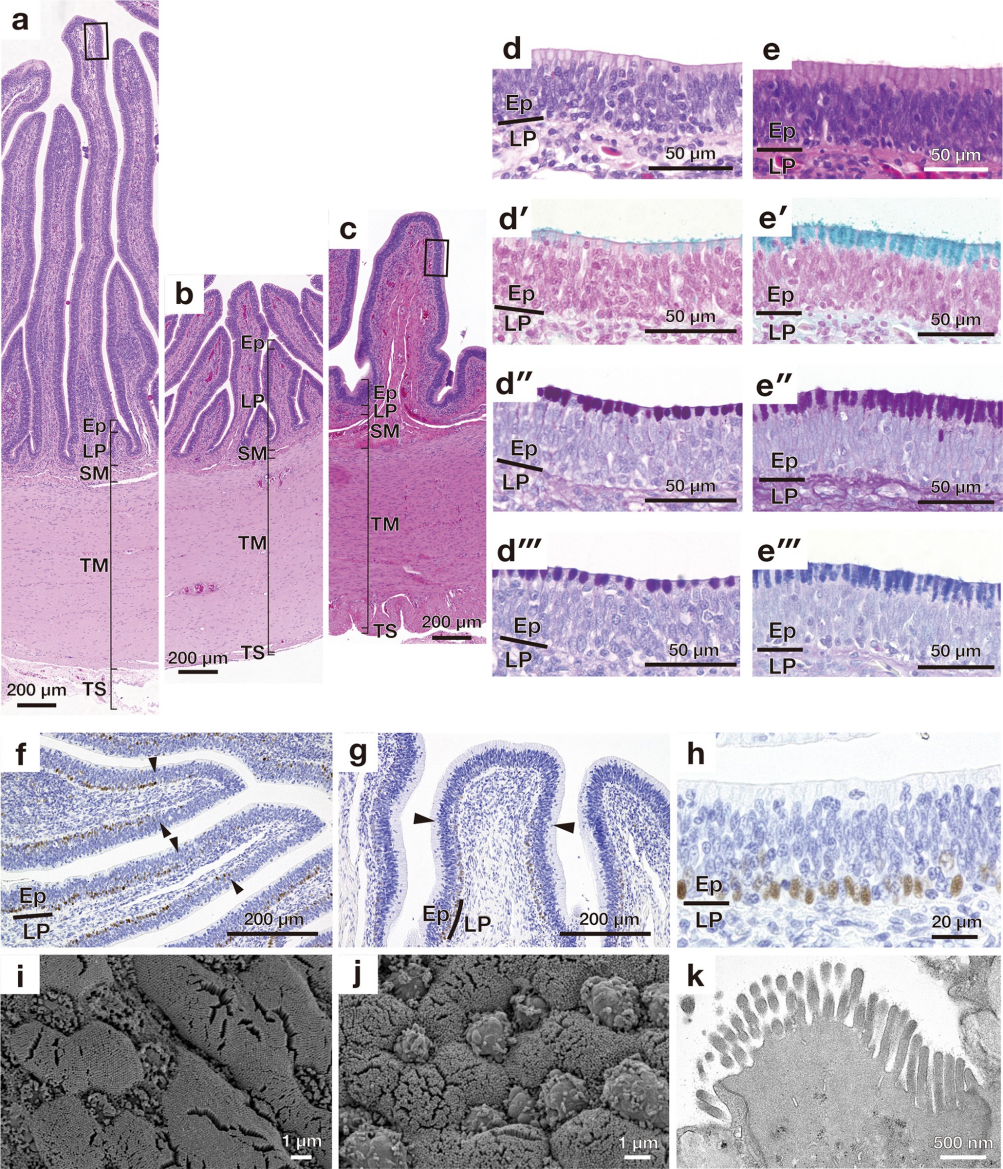
Microscopic appearances of longitudinal folds in the small intestine. Overall cross-sectional view of mucosa stained with haematoxylin and eosin in the proximal (a), middle (b), and distal (c) small intestines. (d) Higher magnification image of the mucosal epithelium enclosed by solid lines in (a). (d′)-(d‴) show high magnification images of the epithelial layer stained with alcian blue (AB) (d′), periodic acid Schiff (PAS) (d″), combined AB and PAS (d‴). (e) Higher magnification image of the mucosal epithelium enclosed by solid lines in (c). (e′)-(e‴) show high magnification images of the epithelial layer stained with alcian blue (AB) (e′), periodic acid Schiff (PAS) (e″), combined AB and PAS (e‴). (f) and (g) show proliferating cell nuclear antigen (PCNA)-positive cells present in the basal part of the epithelium of the proximal (f) and distal (g) small intestine. Arrowheads indicate the boundary between the apical and middle portions of the mucous fold. (h) shows a high magnification image of PCNA-positive cells in the epithelium. (i) and (j) are scanning electron microscopy images of the mucosal epithelium of the proximal (i) and distal (j) small intestine. (k) Transmission electron micrograph of enterocyte having microvilli. Ep, epithelium; LP, lamina propria; SM, submucosa; TM, tunica muscularis; TS, tunica serosa.

**Fig 8:**
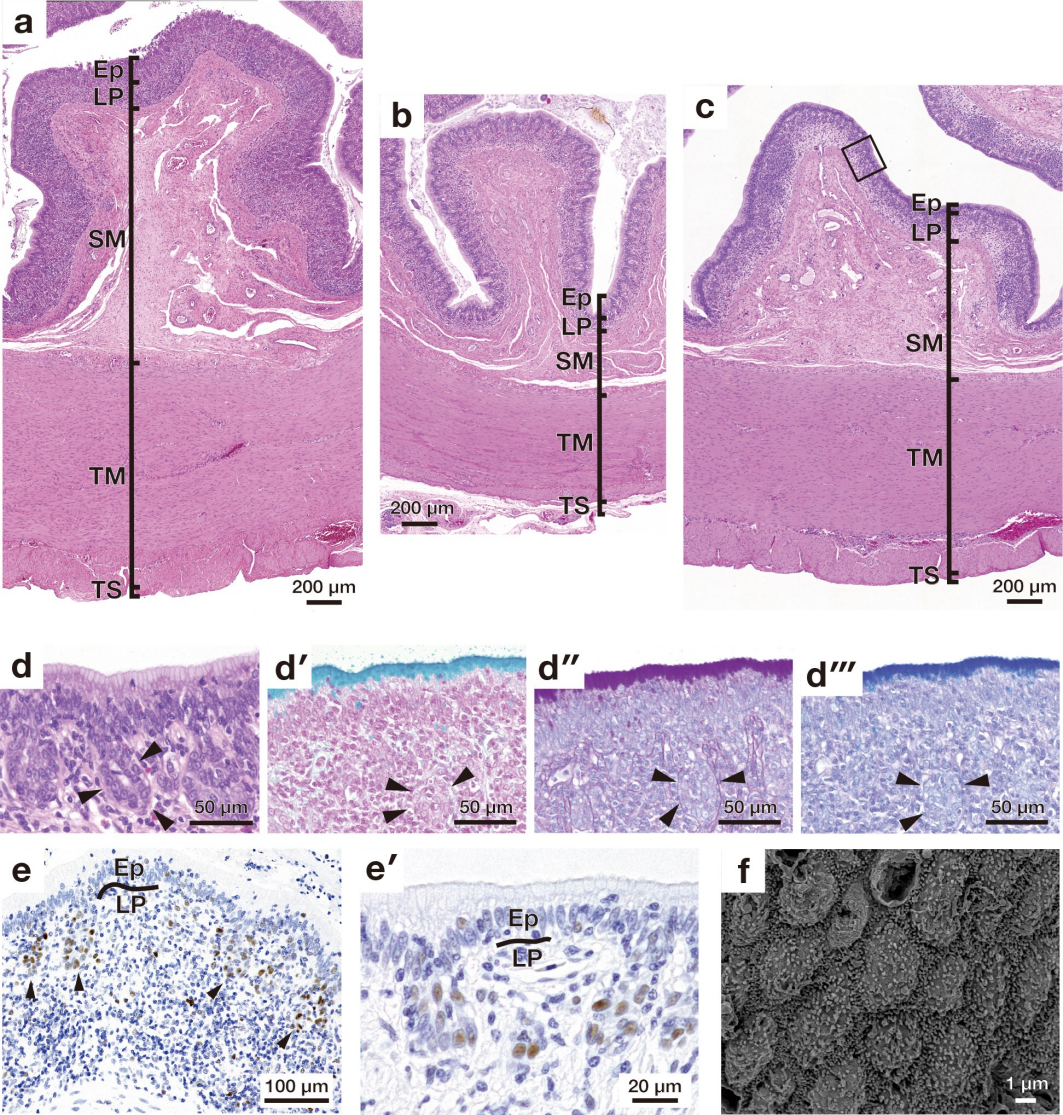
Microscopic appearance of mucosal folds in the large intestine. (a)-(c) show the overall cross-sectional views of mucosa stained with haematoxylin and eosin in the cecum (a), proximal large intestine (b), and distal large intestine (c). (d) Higher magnification image of the mucosal epithelium enclosed by solid lines in (c). Arrowheads indicate the crypt-like structure. (d′)-(d‴) show high magnification images of the epithelial layer stained with alcian blue (AB) (d′), periodic acid Schiff (PAS) (d″), combined AB and PAS (d‴). Arrowheads indicate the crypt-like structure. (e) shows proliferating cell nuclear antigen (PCNA)-positive cells present in the crypt-like structure. (e′) shows a high magnification image of PCNA-positive cells. (f) Scanning electron micrograph of the mucosal epithelium of the large intestine. Ep, epithelium; LP, lamina propria; SM, submucosa; TM, tunica muscularis; TS, tunica serosa.

In all segments of the digestive tract, the walls consisted of mucosa, submucosa, tunica muscularis, and tunica adventitia or serosa (Figs 3–8). The mucosa consisted of the epithelium, lamina propria, and lamina muscularis mucosae, and the mucosal folds comprised mucosa and submucosa. Except for the body of stomach, the mucous cells were stained with both AB and PAS (Figs 3 to 8). Mucous cells were uniformly distributed from the apical to the basal part of the longitudinal folds in all segments. The lamina propria and submucosa were layers of connective tissue containing abundant collagenous fibres, vessels, and smooth muscle fibres. Collagenous fibres in the submucosa were looser than those in the lamina propria, and the tunica muscularis consisted of two smooth muscle layers: thick inner circular and thin outer longitudinal layers (Figs 3–8). The serosa, a simple squamous epithelium, covered all segments of the digestive tract, except for the esophagus, which was covered with tunica adventitia (Figs 3–8). Each layer of the digestive tract showed a segment-specific morphology. The segment-specific characteristics in the mucous cell distribution and wall structure are described below.

### Esophagus

The esophagus was lined by the pseudostratified epithelium consisting of ciliated, goblet, and basal cells (Fig 3B). The goblet cells stained with both AB and PAS and the ciliated cells were located at the superficial layer of the pseudostratified epithelium (Fig 3B′–3B‴). In the luminal surface of the esophagus, goblet cells were not detected, and it was covered by long cilia (Fig 3C and, 3D). The basal cells of the pseudostratified epithelium contained PCNA-positive proliferating cells (Fig 2E and 2E′). At the esophago-gastric junction, the epithelium was composed of ciliated, mucous, and basal cells, as in the esophagus; however, the height of the epithelial cells was lower than that of the esophagus (Fig 3F). Both ciliated and goblet cells were detected in the luminal surface of the esophago-gastric junction (Fig 3G).

### Stomach

The luminal surface of the cardia, body of the stomach, and pylorus was lined by simple columnar epithelium consisting of surface mucous cells (Figs 4B, 5B and 6B). The gastric pit was detected in all three regions of stomach, and those in pylorus had relatively deep gastric pits in comparison with the cardia and body of the stomach (Figs 4A, 5A, and 6A). In the cardia and pylorus, the surface mucous cell, cardiac gland, and pyloric gland are stained with both AB and PAS (Figs 4B′–4B‴ and 6B′–6B‴). Also, in the body of the stomach, the surface mucous cells, mucous neck cells, and mucous cells in the gastric glands were stained with PAS (Fig 5B′ and 5C′), however, only in gastric pits and neck of the gastric gland, the mucous cells were stained with AB weakly (Fig 5B′–5B‴, 5C′–5C‴). PCNA-positive proliferating cells were present in the neck of the proper gastric gland in all segments of the stomach (Figs 4C, 4C′, 5D, 6C, 6C′). In the body of stomach, abundant gastric glands composed of oxynticopeptic, and mucous cells were observed in the lamina propria (Fig 5C). Oxynticopeptic cells had secretory granules, intracellular canaliculi, and abundant mitochondria (Fig 5F).

### Small intestine

In the proximal, middle, and distal small intestines, the epithelium was the pseudostratified columnar epithelium consisting of absorptive epithelial, goblet, and basal cells (Fig 7D and 7E). The goblet cells stained with both AB and PAS lined the luminal surface of the longitudinal folds (Fig 7D′–7D‴, 7E′–7E‴). In comparison with the proximal and middle small intestine, the distal small intestine had a greater number of goblet cells which contained larger amount of the mucus (Fig 7D′–7D‴, 7E′–7E‴). In the basal-to-middle portion of the mucous folds, the basal cells contained PCNA-positive proliferating cells, whereas no PCNA-positive proliferating cells were found at the apical portion of mucosal folds (Fig 7F–7H). The microvilli and goblet cell were detected in the luminal surface of the small intestine (Fig 7I–7K), and the lamina propria in the longitudinal folds contained abundant capillaries (Fig 7A–7C). The outer longitudinal layer of the tunica muscularis was thin in the proximal and middle small intestines, but thick in the distal small intestine (Fig 7A–7C).

### Large intestine

All large intestinal segments showed similar morphological features in each layer (Fig 8A–8C). The luminal surface of the large intestine was lined by the simple columnar epithelium consisting of numerous goblet cells, and the crypt included PCNA-positive proliferating cells (Fig 8D–8D‴, 8E–8F). In the large intestine, the mucous cell stained with both AB and PAS lined the lumina surface of the longitudinal fold (Fig 8D′–8D‴).

## Discussion

**Fig 9:**
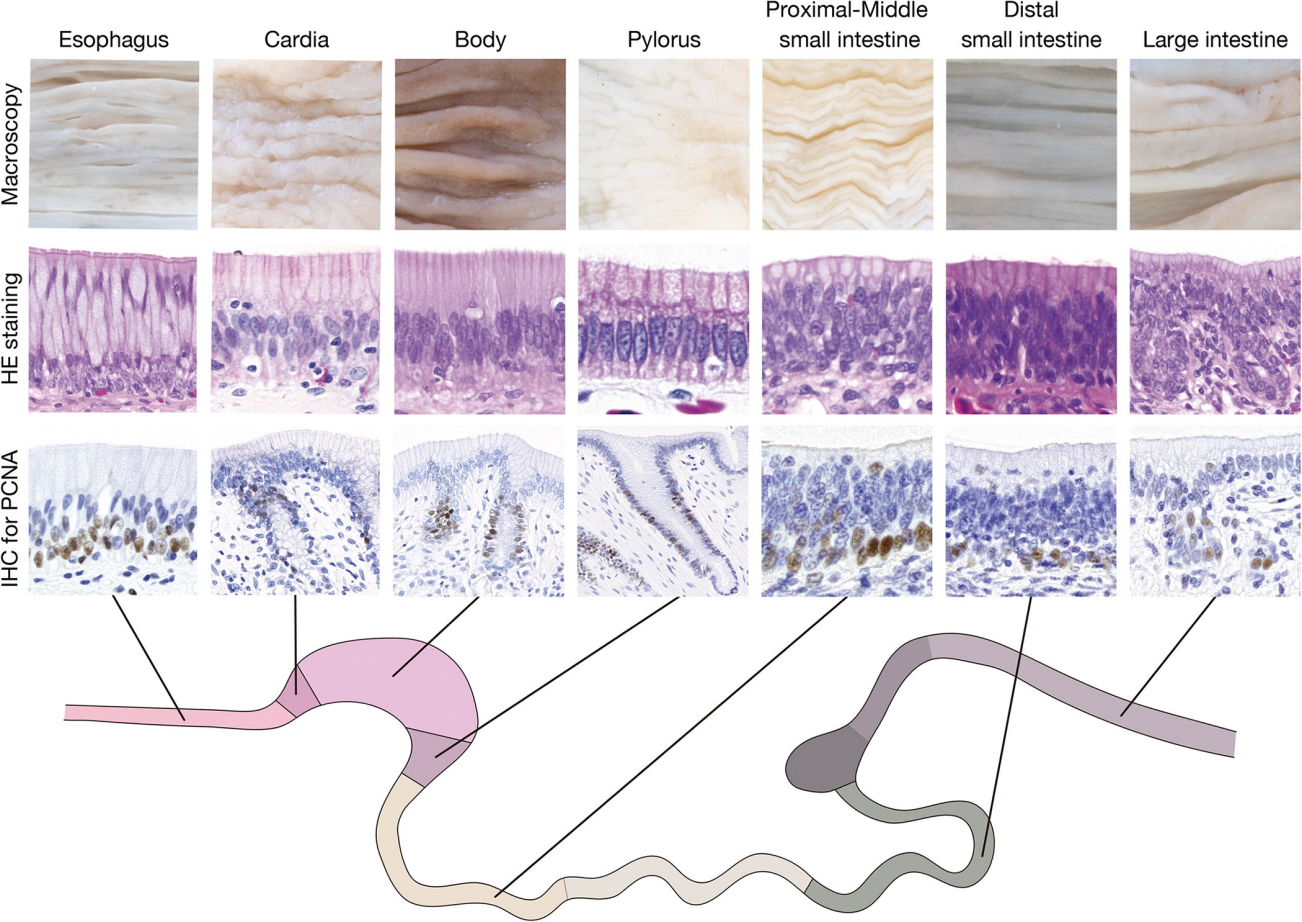
Graphical summary of the findings of the present study. Upper, middle, and lower lines show the macroscopic morphology of the mucosal folds, micrographs of epithelium stained with hematoxylin an eosin, and micrographs of proliferating cell nuclear antigen-positive cell in each segment of digestive tract, respectively. HE, hematoxylin an eosin; IHC, immunohistochemistry; and PCNA, proliferating cell nuclear antibody.

This is the first study to comprehensively describe the macro- and microscopic morphology of the digestive tract in red-eared sliders. Main findings in the present study are summarized in Fig 9. The novel and important findings of the present study are summarised in 1) variations in the longitudinal fold morphology along the oro-anal axis of the digestive tract, and 2) detailed cytoarchitecture of the mucosal epithelium lining the digestive tract.

### Segmental and species differences in the mucosal fold morphology of the digestive tract

To understand our findings in the present study, we summarized previously reported anatomical characteristics in the digestive tract of turtles. As shown in Table 1, the internal appearance of the digestive tract in turtles shows a variety of morphologies depending on each segment and species. The longitudinal folds are commonly detected in most segments of the digestive tract in many species [13–16, 18, 26]. Moreover, intestinal villi are described only in the Indian tent turtle (Pangshura tentoria) [27], as observed in mammals; however, we did not see any villa-like structures in the small intestine of red-eared sliders. The presence of longitudinal mucosal folds and absence of intestinal villi in the digestive tract lumen, as observed in red-eared sliders, is expected to represent the basic structure of turtles.

**Table 1:** Internal appearance of each intestinal segment in each turtle species.

Suborder	Species	Morphology of the mucosal fold in each intestinal segment								
		Esophagus	Stomach		Small intestine			Cecum	Large intestine	
			proximal	distal	proximal	middle	distal		proximal colon	distal colon
Cryptodira	Red-eared slider (present study)	longitudinal	longitudinal		zigzag-longitudinal		longitudinal	longitudinal	longitudinal	
	Egyptian tortoise (Tahon et al. 2021)				-			misshapen	misshapen	
	Greek tortoise (Perez-Tomas et al. 1990)				longitudinal			longitudinal	longitudinal	
	Green sea turtle (Magalhãesl et al. 2012)	papillae			reticular	longitudinal		smooth	longitudinal	
	Green sea turtle (Chen et al. 2015)		longitudinal	transverse	zigzag-longitudinal			longitudinal	longitudinal, transverse	longitudinal
	Leatherback turtle (Magalhãesl et al. 2012)		longitudinal		honey-combed			irregular	irregular	
Pleurodira	Arrau turtle (Magalhãesl et al. 2014)	papillae, longitudinal	transverse	longitudinal	reticulate folds	zigzag-longitudinal		transverse	longitudinal	
	Yellow spotted river turtle (Magalhãesl et al. 2014)									
	Red-headed amazon river turtle (Magalhãesl et al. 2014)									
	Six-tubercled amazon river turtle (Magalhãesl et al. 2014)		longitudinal					no orientation	no orientation	
	Big-headed amazon river turtle (Magalhãesl et al. 2014)									
	Vanderhaege’s toad-headed turtle (Pinheiro et al. 2010)	smooth, longitudinal			zigzag-longitudinal			-	longitudinal	

There are a few detailed descriptions of changes in the mucosal fold structure along the oro-anal axis within the segment of the turtle digestive tract. In the small intestine of red-eared sliders, the shape of the mucosal folds changed along the oro-anal axis of the digestive tract. In turtles without intestinal villi, the longitudinal mucosal folds increase the area of contact with the contents of the digestive tract, and changes in their morphology along the oro-anal axis of the small intestine may attribute to the segmental difference in absorptive efficiency of the nutrients. Moreover, in the esophagus, the mucosal fold morphology of the freshwater and terrestrial turtles is clearly different from that in the marine turtle. The esophageal mucosal fold may be adapted to their respective environments in contact with freshwater, seawater, and air.

### Segmental and species differences in the cytoarchitecture of the epithelium

Table 2 summarises the epithelial structure of the digestive tract in each turtle species. Previous studies on tortoises, sea turtles, and freshwater turtles have shown that the mucosal epithelium of the stomach, small intestine, and large intestine commonly have a simple columnar structure [14–16]. In the stomach and large intestine, the red-eared slider showed an epithelial structure similar to that of other turtles [14–16, 25, 27, 28]. In contrast, the small intestine was lined by the pseudostratified epithelium, which is different from most of other turtle species [14–16, 25, 27, 28]. Moreover, the esophageal epithelium is clearly different among species [14–16, 29]. In many turtle species, the esophagus is lined by a stratified squamous epithelium, whereas the esophagus of red-eared sliders has the pseudostratified epithelium composed of the goblet cell, ciliated cell, and basal cell. This type of epithelium is generally found in the trachea-bronchial epithelium, and carries foreign bodies and micro-organisms trapped in mucus into the upper respiratory tract [30]. The function of the ciliated epithelium of the esophagus remains unknown but might be similar to that of the airways, with a higher ability to prevent foreign hazard materials entering the stomach. Large amounts of mucus produced with mucinous cells might also help a variety of foods move into the stomach.

**Table 2:** Epithelial structure of each intestinal segment in each turtle species.

Suborder	Species	Morphology of the epithelium					
		esophagus	stomach			small intestine	large intestine
			cardia	body	pylorus		
Cryptodira	Red-eared slider (present study)	pseudostratified ciliated columnar	simple columnar			pseudostratified columnar	simple columnar
	Indian tent turtle (Rahman and Sharma 2014)	simple ciliated columnar	-			simple columnar	simple columnar
	Egyptian tortoise (Tahon et al. 2021)	non-keratinized stratified squamous	simple columnar			simple columnar	
	Greek tortoise (Perez-Tomas et al. 1990)	non-keratinized stratified squamous, stratified columnar				-	
	Green sea turtle (Chen et al. 2015)	keratinized stratified squamous	stratified squamous, stratified columnar, simple columnar	simple columnar		simple columnar	
	Olive ridley sea turtle (Yoshie and Honma 1976)		-			-	-
	Loggerhead sea turtle (Tlachi et al. 2014)	-	-			pseudostratified columnar	-
Pleurodira	Arrau turtle (Magalhãesl et al. 2014)	non-keratinized stratified squamous, stratified columnar	simple columnar			simple columnar	simple columnar
	Yellow spotted river turtle (Magalhãesl et al. 2014)						
	Red-headed amazon river turtle (Magalhãesl et al. 2014)						
	Six-tubercled amazon river turtle (Magalhãesl et al. 2014)						
	Big-headed amazon river turtle (Magalhãesl et al. 2014)						

The glandular cells in the proper gastric gland of turtles have been inconsistently described [14–16]. Using optical microscopy, Chen et al. and Tahon et al. reported that glandular cells in the proper gastric gland could not be distinguished between chief and parietal cells in green sea turtles and Egyptian tortoises [14, 15]. Perez-Tomas et al. detected oxynticopeptic cells in the proper gastric gland of Greek tortoises using TEM [16]. Our observations also showed that optical microscopy could only distinguish between glandular and mucous cells of the gastric glands, and TEM observation was required to identify oxynticopeptic cells. It is assumed that the proper gastric gland of turtles is composed of oxynticopeptic cells instead of chief and parietal cells, probably when observed with optical microscopy and electron microscopy.

In the red-eared sliders examined here, the distribution patterns of PCNA-positive proliferating cells differed in each segment. In the stomach and large intestine, PCNA-positive cells were detected in the neck of the proper gastric gland and in crypt-like structures, respectively. The distribution of proliferating cells in the stomach and large intestine is similar to that in mammals [31, 32]. However, in the esophagus and small intestine in red-eared sliders, the distribution pattern of these cells is different from that in mammals [31, 32]. As to stem and progenitor cells, the esophagus and small intestine revealed the basal cell type system as observed in the epidermis, not the crypt base system. The basal cell system can send functionally differentiated cells directly toward the entire mucosal surface and might rapidly renew epithelial cells to maintain homeostasis. The crypt base system can send functional differentiated cells slowly over time from the depths of the crypts to the mucosal surface; however, the system does not fully develop in red-eared sliders, as clear crypts like mammalian type crypts were not observed in the large intestine. The biological differences between these two systems on mucosal homeostasis and function (absorption of nutrition) remain uncertain; however, rapid and slow renewal systems might be specifically maintained in red-eared sliders’ digestive tract. It is conceivable that the epithelial cell dynamics are different between mammals and turtles, but limited knowledge is available on the distribution of proliferating cells in the digestive tract of turtles. Future detailed analyses, including stem cell phenotypes, are required to provide clues for understanding the mechanisms for epithelial maintenance in turtles.

### Limitations of this study

The physiology of turtle digestive tract is not fully understood. Future studies for physiology of the turtle digestive tract are required to understand the functional significance of the segmental difference in the mucosal fold and epithelial structures. Endoscopic examination and a high-speed camera observation may reveal the motility of the mucosal fold and cilia in the esophagus. Moreover, an experimental approach to mucosal injury and regeneration might be required to understand the importance of the characteristic proliferating cell distribution in each digestive tract segment. Cell tracing techniques may be useful to elucidate the segment difference in the epithelial cell dynamics. Furthermore, as the epithelial cells showed a high density, the identification between the stratified epithelium and pseudostratified epithelium was difficult with microscopic observation. Considering the function of the ciliated cell, goblet cell, and absorptive cell, the epithelium was determined as the pseudostratified epithelium in the esophagus and small intestine in this study. Although a limited degree of discussion is possible based on the morphological findings alone, we believe that our study provides an anatomical basis for a novel approach to understand the evolution of the digestive tract.

### Conclusions

This study revealed segmental differences in the longitudinal mucosal fold morphology along the oro-anal axis of the digestive tract and a detailed cytoarchitecture of the mucosal epithelium in the red-eared slider. The findings of the present study enhance our knowledge of the comparative anatomical feature of the digestive tract of water and marine turtles, some of which have a high adaptability to a variety of ecological environments and can eat various food, like red-eared sliders, but a low adaptability to a limited ecological environment and can eat only limited food, like sea turtles.
